# Azo Dye Biodecolorization Enhanced by *Echinodontium taxodii* Cultured with Lignin

**DOI:** 10.1371/journal.pone.0109786

**Published:** 2014-10-06

**Authors:** Yuling Han, Lili Shi, Jing Meng, Hongbo Yu, Xiaoyu Zhang

**Affiliations:** College of Life Science and Technology, Huazhong University of Science and Technology, Wuhan, China; Universidade Nova de Lisboa, Portugal

## Abstract

Lignocellulose facilitates the fungal oxidization of recalcitrant organic pollutants through the extracellular ligninolytic enzymes induced by lignin in wood or other plant tissues. However, available information on this phenomenon is insufficient. Free radical chain reactions during lignin metabolism are important in xenobiotic removal. Thus, the effect of lignin on azo dye decolorization in vivo by *Echinodontium taxodii* was evaluated. In the presence of lignin, optimum decolorization percentages for Remazol Brilliant Violet 5R, Direct Red 5B, Direct Black 38, and Direct Black 22 were 91.75% (control, 65.96%), 76.89% (control, 43.78%), 43.44% (control, 17.02%), and 44.75% (control, 12.16%), respectively, in the submerged cultures. Laccase was the most important enzyme during biodecolorization. Aside from the stimulating of laccase activity, lignin might be degraded by E. taxodii, and then these degraded low-molecular-weight metabolites could act as redox mediators promoting decolorization of azo dyes. The relationship between laccase and lignin degradation was investigated through decolorization tests in vitro with purified enzyme and dozens of aromatics, which can be derivatives of lignin and can function as laccase mediators or inducers. Dyes were decolorized at triple or even higher rates in certain laccase–aromatic systems at chemical concentrations as low as 10 µM.

## Introduction

More than 700,000 tonnes of dyes are produced annually [1]. Azo, anthraquinone, sulfur, indigoid, triphenylmethyl (trityl), and phthalocyanine are most commonly used in industries, but azo derivatives account for approximately 70% of all synthetic colorants [2]. Processing and daily washing release up to 15% of the used dyes into the aquatic environment [3]. This practice adversely affects the aesthetic value of water bodies and poisons aquatic and terrestrial organisms [4]. All synthetic dyestuffs have chemical structures resistant to light, water, chemicals (e.g., oxidizing and reducing agents), and biological corrosion, making effluents from these compounds refractory pollutants that notably affect conventional biological wastewater treatments [5], [6]. Traditional biological treatments of synthetic dyes such as activated sludge and biofilm are inexpensive and green alternatives. However, the efficiency of these techniques in the treatment of textile effluents considerably varies. Thus, a more economical and effective approach to dispose of wastewater with an extensive variety of dyes should be explored and implemented.

White-rot fungi (WRF) have attracted increasing scientific attention in environmental pollutant removal because of their unique ligninolytic enzyme system. Those enzymes are mainly extracellular and exhibit broad substrate specificity to decompose the complex and random phenylpropanoic polymeric structure of lignin [7]. Bioprocessing of dyes using certain WRF strains cultured under optimal fermentation conditions programmed for higher ligninolytic enzyme production have been investigated. Given the importance of extracellular enzymes in biodegradation, decolorization using crude or pure enzymes has been extensively explored. However, whole cells are superior to crude enzymes and much better than purified enzymes in generating nontoxic, harmless, or even completely mineralized end products; thus, dye decolorization significantly depends on an integrated fungal working system [8]. Hence, whole-cell treatment systems are further investigated for better utilization. Stains were found to fade out more rapidly in WRF cultures with low-cost lignocellulose substrates, comprising a wide variety of wastes from agricultural, forest, and food industries. Lignin-degrading enzymes were generally observed to be induced by natural additives than by synthetic media [9]–[13]. However, the mechanism behind the simultaneous increase in decolorization and enzyme production in natural substrates has not been elucidated. The biodegradation enhancement has been perceived to have resulted from the added oxidoreductases caused by the complex carbon resources or from the phenolic extracts from lignocellulose. Ligninolytic enzymes are generally promoted by nutrient structure (especially nitrogen limitation) regardless of the existence of a pollutant [8]. Moreover, phenols in lignocellulose can easily be released and participate in the enzymatic reaction as cofactors or redox mediators [8], [14] or in the activation and induction of ligninolytic enzyme. In addition, lignin extracted from effluents of pulping and papermaking industries was often used as a superior stimulator for ligninolytic enzyme production [15].

However, discussions on the improvement of decolorization using WRF grown on lignocelluloses, the mimetism of fungal primitive habitat, are speculations and ambiguous, without any records directly revealing the mechanism behind the lignin/lignocellulose induction of enzymes. Moreover, lignin metabolism during the secondary metabolic stage along with xenobiotic compound degradation is often neglected. Lignin is a highly stable natural heterogeneous polyphenolic biopolymer [16]. Thus, this compound can be selectively decomposed by fungal ligninolytic enzymes, such as low-redox potential laccase and high-redox potential ligninolytic peroxidases. These enzymes can nonspecifically attack the chemical bonds between phenylpropane units through free radical chain reactions and break down the structures of lignin as well as various xenobiotics, especially azo dyes, which share a similar aromatic skeleton with lignin [17], [18]. The oxidative breakdown of lignin phenylpropanoid units releases low-molecular-weight products [19], which may act as stimulators for laccase or ligninolytic peroxidases [13]. Furthermore, humus and some quinone compounds, e.g., anthraquinone-2,6-disulfonate, which can be transformed from lignin by WRF [20], can function as redox mediator and significantly accelerate the biotransformation of azo dyes, nitroaromatics, or chlorinated aromatics [21]. Thus, when pollutants are degraded by WRF in the presence of lignocellulose, the metabolism of lignin (the second most abundant component in lignocellulose) positively participates in aromatic xenobiotic degradation. However, no direct proof for this phenomenon has been obtained, necessitating further investigations. The clarification of this phenomenon will facilitate the control of the bioprocessing of pollutant by WRF cultured with lignocelluloses.

This study was designed to evaluate the relationship between lignin decomposition and the efficiency of azo dye decolorization to determine the mechanism by which lignin metabolism affects aromatic pollutant degradation and provide guidance to the biological treatment of xenobiotics. The physicochemical properties and enzymatic activities of extracellular fluid were investigated in this study. Possible lignin metabolites and their function were determined by purifying and inoculating the key ligninolytic enzyme involved in biodegradation using dyes and various lignin-derived compounds.

## Materials and Methods

### Ethics statement

No specific permissions were required for Shen-nung-chia Nature Reserve (Hubei Province, P.R. China) and the location was a scenic spot. The field studies did not involve endangered or protected species and the fungus was common in this location. Echinodontium taxodii 2538 was reported in our previous studies [22]–[25] and the GenBank accession number of this fungus was EF422215.

### Microorganism and growth conditions

*Echinodontium taxodii* (GenBank accession number, EF422215) was isolated from rotten wood in Shen-nung-chia Nature Reserve (a scenic spot in Hubei Province, P.R. China) and preserved by our laboratory [24]. This strain was chosen for the present study because of the following reason. In our previous researches on biological pretreatment of lignocelluloses for energy production, the fungus exhibited great selective lignin-degrading ability [22]–[25]. Besides, *E. taxodii* was able to decolorize various dyestuffs in a pretest of environment pollutants removal. The organism was maintained on maize straw plate at 4°C with periodic transfer and activated on basic medium agar plate at 28°C for 7 days [26].

### Dyestuffs and chemicals

Remazol Brilliant Violet 5R (RBV5), Direct Red 5B (DR5B), Direct Black 38 (DB38) and Direct Black 22 (DB22) were purchased from Aladdin. 2,2′-Azinobis,3-ethylbenzothiazoline-6-sulfonic acid (ABTS) and lignin (CAS Number: 8068-05-1, Product Number: 370959) were obtained from Sigma-Aldrich. Veratryl alcohol and other fine chemicals were bought from Sinopharm Chemical Reagent Co. Ltd. China.

### Effect of lignin on azo dye decolorization by *Echinodontium taxodii*

Three agar plugs with 1 cm diameter, which were punched from the periphery of 7-day agar plates, were cultivated in a stationary 250-mL flask containing 30 mL medium at 28°C. Quadruplicate flasks were used for each group. The media components of the four groups for each dye set in this study are shown in Table 1. Dye groups were used as the control for lignin–dye groups. Media containing only lignin were set to examine the effect of lignin on *E. taxodii*, which was inoculated in basic media as the corresponding control.

**Table 1:** The media used in this study.

contents	control	lignin	dye	Lignin-dye
basic medium	+	+	+	+
lignin	−	+	−	+
dye	−	−	+	+

+ With the content listed on the left added

− Without the content listed on the left added

The basic medium (per liter distilled water) contained 10.0 g glucose, 0.2 g ammonium tartrate, 2 g KH_2_PO_4_, 0.71 g MgSO_4_, 0.1 g CaCl_2_, and 20 mL trace element solution. The trace element solution had the following composition (per liter distilled water): 1 g NaCl, 0.5 g MnSO_4_·H_2_O, 0.1 g CoCl_2_, 0.1 g FeSO_4_·7H_2_O, 0.1 g ZnSO_4_·7H_2_O, 0.1 g CuSO_4_·5H_2_O, 0.01 g H_3_BO_3_, 0.01 g Na_2_MoO_4_·2H_2_O, and 0.01 g KAl(SO_4_)_2_·12H_2_O. The media were prepared as 1.2-fold-concentrated stock solutions and 25 mL of the media were split into 250-mL flasks. Azo dyes (RBV5, DR5B, DB38 and DB22, additional information on these dyes are shown in Table 2) were dissolved in distilled water with a final concentration of 600 mg/L. Then, distilled water, the media and dyes were autoclaved at 115°C for 20 min. After that, dye solutions or distilled water (5 mL) were added to each flask sterilely. Different concentrations of lignin (0.1, 0.3, and 0.5 g/L) were sterilized with ultraviolet light for 30 min before being added to the sterilized media. Considering the disoriented state of water-soluble components, lignin was washed thoroughly with distilled water and then freeze-dried to constant weight before use. The cultures of each group in quadruplicate flasks were taken on the day after inoculation. The fermentation liquor was obtained by filtering out the mixture of mycelia and lignin residues before centrifugation at 10,000×*g* for 10 min to remove water-insoluble lignin. The mycelia were oven-dried to a constant weight and were calculated as the biomass after the solid mixture was washed through a 100-mesh filter gauze to remove the lignin and media residues.

**Table 2:** Characteristics of the synthetic dyes studied in this work.

Dyes	CAS	Molecular Formula	λ_max_(nm)	Number of azo bonds
RBV5	12226-38-9	C_20_H_16_N_3_Na_3_O_15_S_4_	560	1
DR5B	2610-11-9	C_29_H_19_N_5_Na_2_O_8_S_2_	510	2
DB38	1937-37-7	C_34_H_25_N_9_Na_2_O_7_S_2_	550	3
DB22	6473-13-8	C_44_H_32_N_13_Na_3_O_11_S_3_	482	4

In order to evaluate the adsorption by lignin, different concentrations of lignin were added to media containing dyes sterilely, without inoculation. The samples were taken at the same time with cultures mentioned above, and centrifuged at 10,000*×*g for 10 min to remove water-insoluble lignin. The liquid supernatant was used to determine the decolorization of lignin adsorption.

Decolorization was monitored by measuring the absorbance of the fermentation broth at the maximum wavelength using a spectrophotometer. Decolorization percentage (%) was calculated as follows: decolorization percentage (%) = [(*A*_0_ − *A*)/*A*_0_]×100, where *A*_0_ is the initial absorbance of the dye, and *A* is the absorbance of dye with time.

Laccase activity was determined by measuring the increase in *A*_420_ with 0.5 mM ABTS as the substrate in 50 mM sodium acetate buffer, pH 4.5 (*ε*_420_ = 29,300 M^−1^·cm^−1^) [27]. Manganese peroxidase (MnP) activity was assayed using the method of Wariishi, Valli, Gold [28] and by measuring the increase in absorbance at 270 nm with 1 mM Mn^2+^ as the substrate in 50 mM malonic acid buffer, pH 4.5 (*ε*_270_ = 11,590 M^−1^·cm^−1^). The reaction was started by adding 0.4 mM H_2_O_2_. Lignin peroxidase (LiP) activity was determined using the method of Tien, Kirk [29], with veratryl alcohol as a substrate in 0.1 M sodium tartrate, pH 3.0 (*ε*_310_ = 9,300 M^−1^·cm^−1^). All reactions were performed at room temperature.

### Effect of lignin-related compounds on azo dye decolorization by purified laccase of *E. taxodii*

Laccase was the only ligninolytic enzyme secreted by this strain during the whole dye removal period, and lignin might affect biodecolorization through this key oxidase. Laccase was purified by salting out with (NH_4_)_2_SO_4_, hydrophobic interaction chromatography with Phenyl Sepharose 6 Fast Flow, and ion exchange chromatography with diethylethanolamine (DEAE)–sepharose Fast Flow.

The *E. taxodii* culture medium was passed through a filter paper to remove fungal mycelia. The supernatant was then subjected to a two-step ammonium sulfate precipitation scheme. First, the supernatant was brought to 45% saturation with ammonium sulfate, and the precipitated proteins were removed by centrifugation at 10,000×*g* for 15 min. Then, solid ammonium sulfate was added again, and the fraction precipitating at 45% to 85% saturation was collected by centrifugation. The precipitate was redissolved in 10 mL of 20 mM sodium acetate–acetic acid buffer (pH 6) containing 0.8 M ammonium sulfate. The supernatant was loaded onto a Phenyl Sepharose 6 Fast Flow column (1.6×20 cm) equilibrated with the same buffer. Unbound proteins were removed by washing the column at a flow rate of 1 mL·min^−1^. Decreasing concentrations of (NH_4_)_2_SO_4_ (0.8 M to 0 M) were used to elute the bound proteins, and the collected fractions (5 mL each) were subjected to enzymatic assay and protein measurement (*A*_280_). The active fractions were pooled, dialyzed against 20 mM sodium acetate–acetic acid buffer (pH 6) overnight, and concentrated by PEG 20000. The concentrate was then applied to a DEAE–Sepharose Fast Flow column (1.6×20 cm) pre-equilibrated with the same buffer. Proteins were eluted with a linear gradient of NaCl (0 M to 0.8 M) at a flow rate of 1.5 mL·min^−1^. Subsequently, fractions containing laccase activity were pooled, concentrated, and desalted. Enzyme purity was then confirmed by sodium dodecyl sulfate–polyacrylamide gel electrophoresis (SDS-PAGE).

Dyes were decolorized in a test tube using purified laccase at room temperature. The reaction mixture (3 mL) contained sodium acetate–acetic acid buffer (50 mM, pH 4.8), purified laccase (final concentration of 1 U/mL), and dyes (final concentration of 100 mg/L) in the presence or absence of lignin-related phenols. The reaction was initiated by adding the enzyme solution under mild shaking conditions. The duration of decolorization was determined by measuring the absorbance at the maximum wavelengths with a Mapada™ UV-1600 spectrophotometer. Decolorization was calculated as described previously. A control test containing the same amount of heat-denatured laccase was conducted in parallel. All reactions were performed in triplicate.

To evaluate dye decolorization using different natural mediators, 12 lignin-related phenols as natural mediators were applied in the decolorization of dyes. These natural mediators (final concentration of 1 mM) were incubated in the buffer described previously at room temperature under mild shaking conditions. In order to evaluate the potential interference of the 12 lignin-related phenols as natural mediators on decolorization measurement, another control test without laccase and dyes were set. The results showed that these phenols had no absorption at those wave lengths, so decolorization was calculated as described previously. The decolorization of dyes was calculated after reaction for 2 h. Then, lower concentrations (10, 50, 100, 250 and 500 µM) of the efficient mediators were applied to the buffer under the same conditions mentioned above for further investigation of the efficiency of these mediators. The decolorization of dyes was detected at different time intervals (from 5 min to 240 min).

## Results and Discussion

### Effect of lignin on dye decolorization by *E. taxodii*

**Figure 1:**
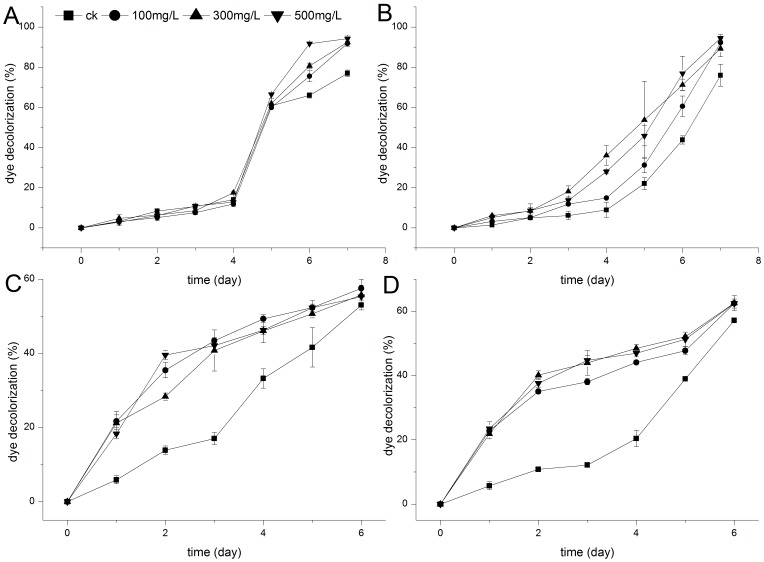
Effect of lignin on the decolorization of different dyes by *E. taxodii.* (A): RBV5. (B): DR5B. (C): DB38. (D): DB22. Dyes concentration: 100 mg/L. Lignin contents: ▪, 0 mg/L; •, 100 mg/L; ▴, 300 mg/L; ▾, 500 mg/L.

Azo reactive dyes RBV5, DR5B, DB38, and DB22 were chosen because they are extensively used in the textile industry worldwide. These azo dyes with 1 to 4 azo groups were tested to establish the relationship between the decolorization effect of *E. taxodii* (with/without lignin) and the structures of azo dyes. *E. taxodii* could remove all four dyes (for as long as 2 wk; data not shown), and the total decolorization percentages of the RBV5, DR5B, DB38, and DB22 controls on day 6 were 65.96%, 43.78%, 53.02%, and 57.20%, respectively. The results showed that the decolorization effect differed from one dye to another, and there was no correlation between the difficulty degree of decolorization and the number of azo bonds. However, compared with the controls, lignin addition enhanced the biological treatment process, no matter how many azo linkages there was (Fig. 1).

In the case of RBV5, *E. taxodii* performed fairly in the first few days. Maximal differences in the decolorization percentage were observed between the control (65.96%) and lignin tests (75.54%, 100 mg/L; 80.64%, 300 mg/L; and 91.75%, 500 mg/L) on day 6, which were 15%, 22%, and 39% higher than that in non-lignin media. On day 7, lignin-free cultures still had dye residues at approximately 25 mg/L, whereas all the test groups had less than 10 mg/L residue. For DR5B, lignin resulted in a notable increase in the decolorization percentage from day 3, and the discrepancies were 5.69% (100 mg/L), 11.95% (300 mg/L), and 7.47% (500 mg/L). On the last day (day 7), control media still showed lower decolorization (75.99%) than the test groups by 16.29% (100 mg/L), 13.37% (300 mg/L), and 18.55% (500 mg/L). In addition, the discrepancies in the decolorization percentage first increased and then decreased along the degradation period. The maximal differences in the decolorization percentage between control and test groups varied, as follows: 100 mg/L, 16.77% on day 6; 300 mg/L, 31.67% on day 5; and 500 mg/L, 33.11% on day 6. For RBV5 and DR5B, the enhancement in decolorization seemed to have intensified with an increase in lignin dosage.

*E. taxodii* in lignin-containing media initially decolorized dye DB38 quickly and then the activity slowed down. However, the fungus worked at nearly constant speed in the blank culture. Different concentrations of lignin did not result in significant differences in dye degradation, but remarkable enhancements were observed when compared with lignin-free experiments in the first few days. Maximum differences in dye decolorization percentage compared with the blank were observed on day 2 or 3 after inoculation, as follows: 26.42% (100 mg/L on day 3), 23.74% (300 mg/L on day 3), and 25.67% (500 mg/L on day 2). Differences gradually decreased, and the control even performed fairly on day 6 (Fig. 1). Differences in decolorization curves caused by lignin indicated that *E. taxodii* treatment of DB38 may follow disparate pathways at first. Similar results were obtained when the fungus reacted with DB22, and more significant promotion than DB38 degradation was recorded. From days 1 to 6, the dye was eliminated much more rapidly along with lignin than the control, and the differences reached maximum values on day 3, as follows: 25.92% (100 mg/L), 31.81% (300 mg/L), and 32.59% (500 mg/L). Afterward, no significant differences were detected.

**Figure 2:**
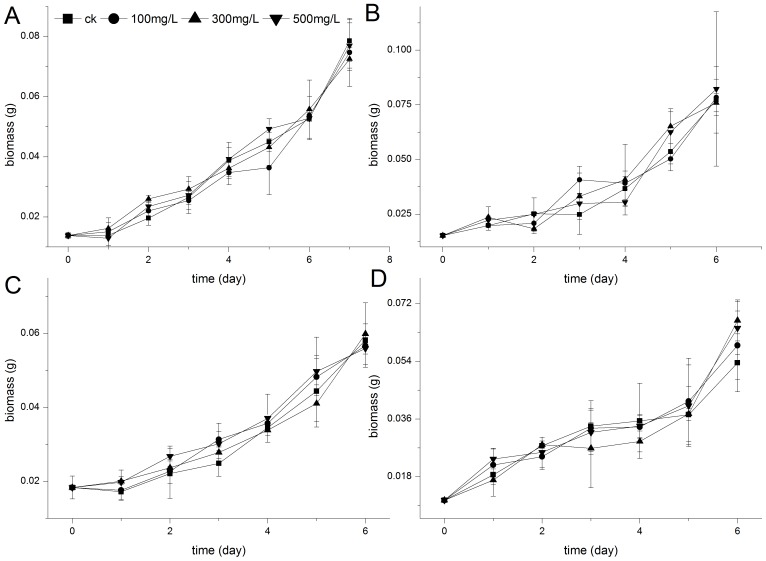
Effect of lignin on biomass during the decolorization of dyes by *E. taxodii*. (A): RBV5. (B): DR5B. (C): DB38. (D): DB22. Dyes concentration: 100 mg/L. Lignin contents: ▪, 0 mg/L; •, 100 mg/L; ▴, 300 mg/L; ▾, 500 mg/L.

Dye bioadsorption using dead or living biomass of WRF has been extensively reviewed in several recent papers [30], [31]. Hence, the absorption function of biomass and lignin was considered in determining the relationship between biological degradation and lignin metabolism. Stationary surface culture was adopted in this study. Results showed that lignin had different absorption patterns on the four dyes. Peak values were all reached on day 2 at the highest lignin concentration with less than 10% absorption as shown in Table 3. In addition, RBV5 and DR5B showed no more than 10% adsorptive decolorization, whereas the actual dye removal was only approximately at 10% independent of the presence of lignin. We observed that lignin was not an ordinary adsorbent for RBV5 and DR5B when cooperating with living fungus. For DB38 and DB22, decolorization differences between lignin–dye groups and the dye controls (>20%) were larger than the highest adsorption values (<10%) on day 2 or 3 (Fig. 1). This result indicated that lignin might enhance dye elimination through some mechanism other than physical–chemistry adhesion. By contrast, biomass from culture inoculated with lignin did not grow faster than that from the control group. However, decolorization seemed to significantly speed up in the former, indicating the absence of difference in the biomass activity on absorption (Fig. 2). This result confirmed that enhanced decolorization was influenced by the metabolism of added lignin.

**Table 3:** Effect of lignin on the adsorptions of azo dyes.

Adsorption Percentage of Azo dyes (%)^a^	Lignin content (mg/L)		
	100	300	500
RBV5	3.72(0.62)	4.31(0.53)	7.21(1.62)
DR5B	3.91(0.78)	6.75(2.09)	9.09(1.02)
DB38	None	None	None
DB22	3.62(0.78)	5.76(1.36)	8.96(1.02)

a Average Percentage with Standard Deviations in Parentheses; N = 3.

### Effect of lignin on ligninolytic enzymes during the decolorization by *E. taxodii*

**Figure 3:**
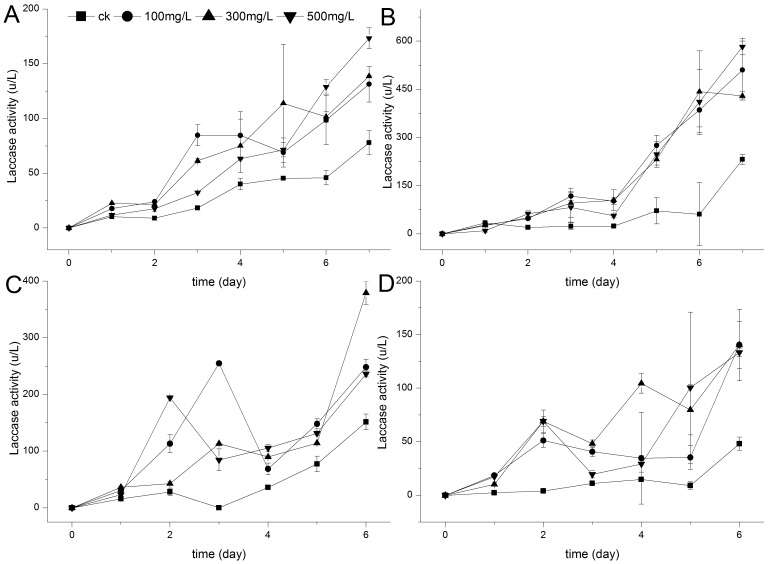
Effect of lignin on laccase activities during the decolorization of dyes by *E. taxodii*. (A): RBV5. (B): DR5B. (C): DB38. (D): DB22. Dyes concentration: 100 mg/L. Lignin contents: ▪, 0 mg/L; •, 100 mg/L; ▴, 300 mg/L; ▾, 500 mg/L.

Fungi degrade lignin or azo dyes through oxidation, probably involving enzymes such as LiP, MnP, and laccase. Meanwhile, toxic aromatic compounds and lignin (lignosulfonate and synthetic lignin) were shown to stimulate the production of laccase, LiP, and MnP [32]. However, in our research, among the main ligninolytic enzymes, only laccase was found in the liquid culture of *E. taxodii* throughout the biological decolorization (Fig. 3). Once lignin was present, significantly higher values of laccase activity were detected in the culture media, even when only lignin was present in the substrate with no dyes. Enzymatic activities were approximately twice to thrice higher than that in the lignin-dye-free blank (<5 IU/L). In addition, dyes alone can stimulate the yield of laccase which generally increased to different extents with the extension of incubation time, as follows: RBV5, 77.95 IU/L; DR5B, 231.92 IU/L; DB38, 53.03 IU/L; and DB22, 48.13 IU/L on the last day of degradation. The stimulation effects of dyes on laccase production during cultivation, which could be regarded as xenobiotic response to detoxification, have also been reported [33]. However, the fungus incubated with lignin produced more laccase and enhanced dye removal (Fig. 3). In the case of RBV5 and DR5B (Figs. 1A, 1B, 3A, and 3B), decolorization percentages were initially not enhanced along with the increase in laccase. Afterward, dyes fade out at higher rates when lignin was added in increasing amounts. However, laccase activities did not occur in similar patterns, i.e., no positive correlation was observed between the two phenomena. For DB38 and DB22 (Figs. 1C, 1D, 3C, and 3D), the decolorization curves were poorly consistent with enzymatic activity detection. All results showed that dyes decolorized in the presence of lignin were affected not only by laccase but also by lignin metabolism. Little information on the combined degradation of pollutants and lignin or the resolution of the inner mechanism is available. According to Tychanowicz, Zilly, de Souza, Peralta [34], structurally different dyes were decolorized because of the high level of laccase generated in response to the presence of soluble phenolic compounds in the glucose/ammonium tartrate–corncob solid-state medium. The fungi produced laccase and removed color at the same time with the supplementation of lignocellulose, providing an alternative approach to deal with textile wastewater and increasing the economic value added by producing laccase [9]. The underlying promoting mechanism should be uncovered to provide a theoretical guideline with strong feasibility in depleting diverse dyes or pollutants.

Thus, Figs. 1 to 3 show that lignin can influence dye removal through laccase, the only oxidase during biotreatment, in some pathway different from the common improvement of enzymatic activity. According to papers published worldwide, WRF secrete a large amount of volatile aromatic compounds, giving rise to natural fragrances, providing a promising source of flavors and aromas. Gutierrez, Caramelo, Prieto, Martínez, Martinez [35] reported that simple aromatic compounds could be generated by fungi with or even without aromatic origins. However, the products varied in quantities and kinds as stimulators were added. Low-molecular-weight aromatic acids have been identified during the long-term degradation of lignin in spruce wood by *Phanerochaete chrysosporium* by high-performance liquid chromatography and after acetylation and methylation by gas chromatography/mass spectrometry (GC/MS) [19]. Additionally, some of these chemicals were low-molecular-weight phenol derivatives and considered potential laccase substrates, which could also work as natural mediators for the oxidation reactions by laccase. Johannes, Majcherczyk [36] tested different kinds of mediators for laccase in the oxidation of polycyclic aromatic hydrocarbons (PAH) and determined that natural compounds such as phenol, aniline, 4-hydroxybenzoic acid, and 4-hydroxybenzyl alcohol are efficient as synthetic chemicals ABTS and 1-hydroxybenzotriazole. Moreover, *p*-hydroxycinnamic acids, namely, *p*-coumaric (4-hydroxycinnamic) acid, ferulic (3-methoxy-4-hydroxycinnamic) acid, and sinapic (3,5-dimethoxy-4-hydroxycinnamic) acid, were used as natural mediators for laccase oxidation of recalcitrant dyes and PAH [37]. Thus, we infer that the metabolites of lignin may act as redox mediators in the oxidation of dyes by laccase, the main oxidative enzyme in decolorization.

In this study, lignin metabolite detection was also conducted. However, we did not observe similar results on the chromatogram map from GC/MS (data not shown) [19]. We infer that lignin degradation in large quantities started during the secondary metabolism of WRF and was a long-term process. Thus, low-molecular-weight lignin derivates were likely in trace amount and were difficult to identify during the short-term decolorization. Moreover, the metabolic intermediates were probably initiated by the increase in laccase during the active growth phase (based on biomass accumulation) when the fungus did not function on lignin degradation. Hence, the metabolic intermediates were difficult to accumulate and often existed briefly. In addition, laccase can graft aromatic substrates back onto lignin [38], [39]. Thus, various lignin-derived aromatics were studied in vitro in mediating degradation by purified laccase.

### Effect of lignin related compounds on the azo dyes decolorization by purified laccase of *E. taxodii*

**Figure 4:**
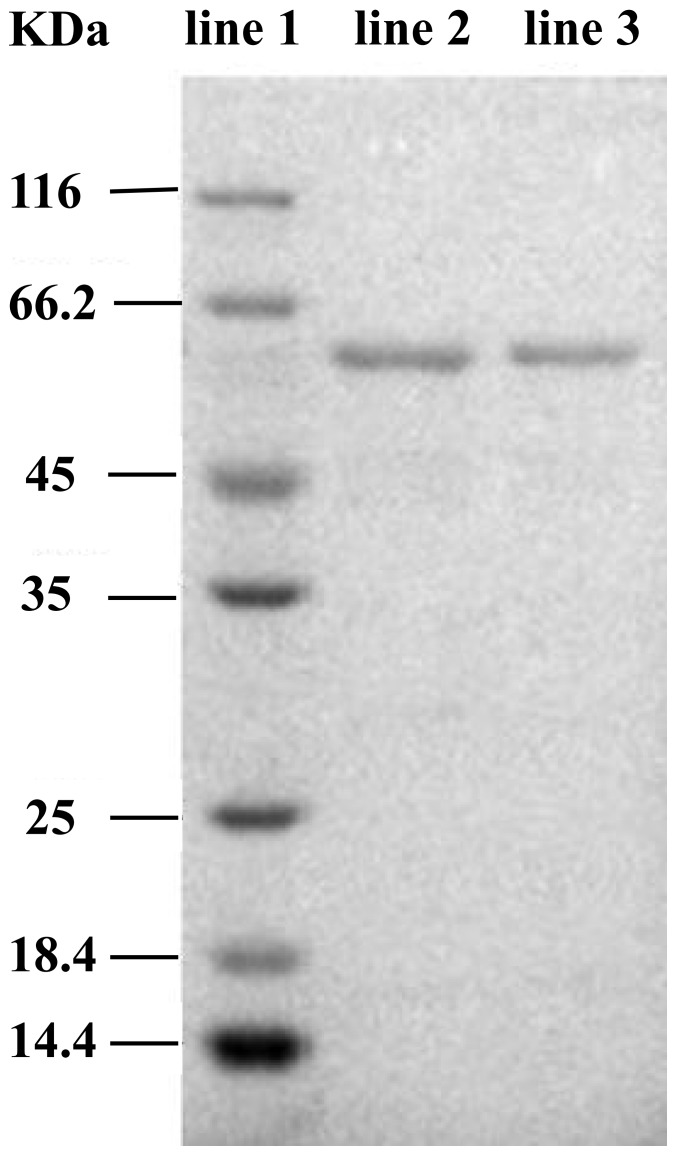
SDS-PAGE analyses of purified laccase of *E. taxodii*. Lane 1, Molecular weight marker; Lane 2 and 3, purified laccase.

Laccase is the most potent oxidase involved in the degradation of lignin and dyes. Laccase was purified from *E. taxodii* cultures to homogeneity by salt fractionation with ammonium sulfate, hydrophobic interaction, and ion exchange chromatography. Approximately 23.3-fold purification was achieved, with an overall yield of 38.8% (data not shown). The purified laccase showed a single protein band on SDS-PAGE (Fig. 4).

**Figure 5:**
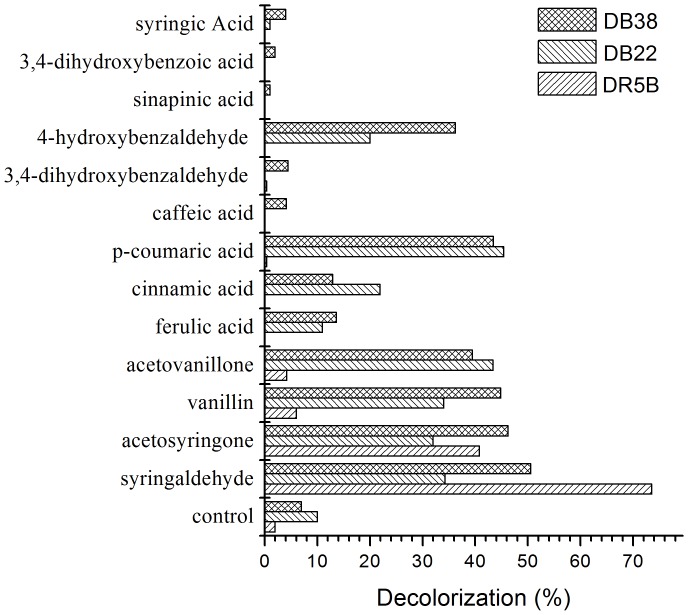
Evaluating for nature mediators based on decolorization of DR5B, DB22 and DB38 (100 mg/L) in presence of mediator (1 mM) for 2 h.

The purified laccase of *E. taxodii* slowly decolorized RBV5, DR5B, DB22, and DB38 in the absence of mediators, with corresponding decolorization percentages of 1%, 2%, 10%, and 7% after 2 h reaction. However, the dye decolorization percentages by laccase were enhanced in the presence of natural mediators, except that of RBV5, which did not disappear more rapidly with mediators present. Among the 13 aromatics (phenolic aldehydes, ketones, and acids), acetosyringone and syringaldehyde significantly promoted the decolorization of DR5B, DB22, and DB38. Decolorization of the last two dyes was also accelerated by *p*-coumaric acid, acetovanillone, and vanillin (Fig. 5). However, we did not obtain any suitable redox mediators in the candidates for RBV5. Among the most efficient mediators, syringaldehyde provided the highest decolorization percentages for DR5B and DB38. The decolorization percentages increased 37-fold and 3.7-fold, respectively, from 2% to 74% for DR5B and from 7% to 51% for DB38. Compared with syringaldehyde, *p*-coumaric acid was more effective in the decolorization of DB22. The decolorization percentage increased 4.5-fold from 10% to 45%. Camarero, Ibarra, Martínez, Martínez [40] determined that syringaldehyde, acetosyringone, vanillin, acetovanillone, methyl vanillate, and *p*-coumaric acid, which were ubiquitous laccase producers of basidiomycetes in decayed wood and forest soils, significantly promoted the oxidation of recalcitrant dyes by laccase.

**Figure 6:**
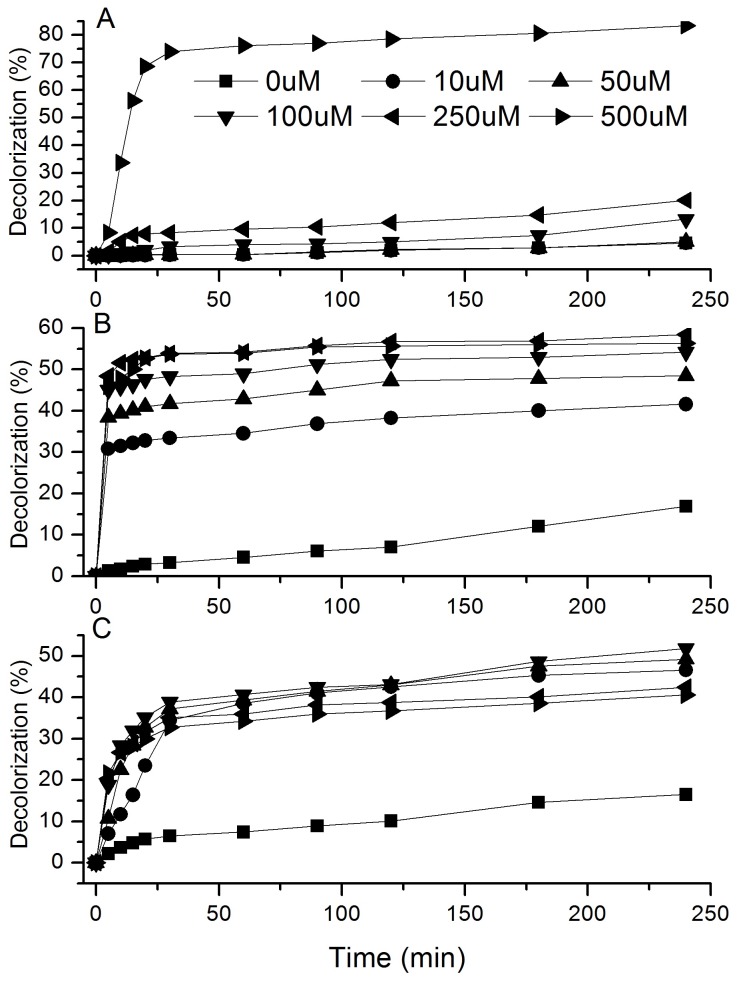
Effects of mediator concentration on dye decolorization (%) by purified laccase (4-h treatment of 100 mg/l DR5B, DB22 and DB38). (A): DR5B + syringaldehyde. (B): DB22 + *p*-coumaric acid. (C): DB38 + syringaldehyde. Mediator concentration: ▪, 0 µM; •, 10 µM; ▴, 50 µM; ▾, 100 µM; ◂, 250 µM; ▸, 500 µM.

The influences of natural mediator concentrations on the decolorization of DR5B, DB22, and DB38 (4 h treatment) are shown in Fig. 6. The decolorization percentages of the three dyes were correlated with the concentration of mediators. Laccase in the presence of *p*-coumaric acid produced a high decolorization percentage for DB22, whereas the presence of syringaldehyde promoted the decolorization percentage for DB38 at a low concentration (10 µM). By contrast, the decolorization percentage for DR5B was directly proportional to syringaldehyde concentration. The addition of syringaldehyde of 1 mM concentration enhanced the decolorization percentage from 0.2% to 74.0% after reaction for 30 min, whereas the decolorization percentage did not change at 10 µM concentration of syringaldehyde.

Although different mediators apparently influence the efficiency of laccase to decolorize the dyes in vitro [40], actual decolorization by *E. taxodii* in the presence of lignin might be affected by the various previously tested lignin-derived compounds. Meanwhile, the contents of each chemical vary from those obtained in this study and were possibly much lower. The result showed that aromatics like p-coumaric acid and syringaldehyde enhanced decolorization by purified laccase, and the enhancement was positively related with the concentration of aromatics. When phenols at a low concentration, they did not increase the decolorization as effectively as they were at a concentration of 1 mM. It was presumed that lignin decomposition product a small quantity of aromatics, and they slowly boosted decolorization in the cultures. By contrast, Chen, Chang, Kirk [19] studied the degradation products of lignin in decayed wood. Monomers and dimers were detected, in which dimers, e.g., dihydroxydivanillic acid and 2′-hydroxy-2,3′-dimethoxydiphenylether, were also produced from monomer polymerization by laccase [41]. Thus, dimers could be detected when aromatics mediated lignin or dye oxidation by laccase. However, natural mediators in the fungal system are not always at a detectable concentration (they are difficult to accumulate and often existed only briefly, but usually performing better than synthetic ones), and the derivatives are in low quantity to be identified, contrary to that in vitro which is set to a much higher level in lignin or xenobiotic compound degradation. Thus, *E. taxodii* degraded lignin and yielded hundreds of metabolites, among which some chemicals worked as redox mediators in laccase oxidation of dyes.

## Conclusions

In the current work *E. taxodii* was observed to degrade four azo dyes more efficiently when lignin was present in the media. Laccase, the dominant enzyme in the biodegradation, was found to be induced by lignin. Experiments in vitro with purified laccase demonstrated that contribution of lignin-related aromatics as laccase mediators might be the explanation for the improvement in decolorization.

## Supporting Information

File S1 Data of Figure 1. Effect of lignin on the decolorization of different dyes by *E. taxodii.* Table S1, RBV5. Table S2, DR5B. Table S3, DB38. Table S4, DB22.
(XLS)

File S2 Data of Figure 2. Effect of lignin on biomass during the decolorization of dyes by *E. taxodii*. Table S5, RBV5. Table S6, DR5B. Table S7, DB38. Table S8, DB22.
(XLS)

File S3 Data of Figure 3. Effect of lignin on laccase activities during the decolorization of dyes by *E. taxodii*. Table S9, RBV5. Table S10, DR5B. Table S11, DB38. Table S12, DB22.
(XLS)

File S4 Data of Figure 5. Evaluating for nature mediators based on decolorization of DR5B, DB22 and DB38 (100 mg/L) in presence of mediator (1 mM) for 2 h. Table S13.
(XLS)

File S5 Data of Figure 6. Effects of mediator concentration on dye decolorization (%) by purified laccase (4-h treatment of 100 mg/l DR5B, DB22 and DB38). Table S14, DR5B. Table S15, DB38. Table S16, DB22.
(XLS)
